# Biofilm-Forming and Multidrug-Resistant *Salmonella* Thompson Strains Isolated from Edible *Cipangopaludina cathayensis*: An Emerging Food Safety Concern in Southern China

**DOI:** 10.3390/microorganisms14102229

**Published:** 2026-10-02

**Authors:** Yuqiong Li, Jianjie Peng, Jie Zhang, Haixia Lan, Ying Wang, Junhao Lin, Shuhui Gan, Xintong Li, Kai Shao, Dapei Zhang, Xiaolu Liang, Ziheng Xu

**Affiliations:** 1 Guangxi Key Laboratory of Translational Medicine for Treating Infectious Diseases with Integrative Medicine, School of Public Health and Management, Guangxi University of Chinese Medicine, Nanning 530200, China; 2 Microbiology Laboratory, Beihai Center for Disease Control and Prevention, Beihai 536006, China

**Keywords:** *Salmonella* Thompson, antimicrobial resistance, biofilm formation, bacterial pathogenicity, *Cipangopaludina cathayensis*, gut microbiota, metabolomics

## Abstract

*Salmonella enterica* Thompson (*S.* Thompson) is a zoonotic foodborne pathogen causing infection in humans and animals, yet limited data exist on isolates from edible *Cipangopaludina cathayensis* (*C. cathayensis*), a key ingredient of Liuzhou Luosifen. In the present study, seven *S.* Thompson isolates recovered from edible *C. cathayensis* in southern China (2021–2022) were characterized in terms of antimicrobial resistance, biofilm formation, resistance and virulence *genes*, and pathogenicity in mice. All seven isolates showed resistance to at least one antimicrobial agent for which valid interpretive criteria exist. Since no Clinical and Laboratory Standards Institute (CLSI) interpretive criteria exist for vancomycin, clindamycin, lincomycin, erythromycin, penicillin, and oxacillin, these agents were excluded from multiple drug resistance (MDR) classification; after reclassification, two isolates, 2/7 (28.6%), fulfilled the criteria for MDR. Five isolates, 5/7 (71.4%), formed biofilms in vitro and carried *csgA* and/or *csgD*; the two non-biofilm-forming isolates lacked both *genes*. All isolates harbored *bla_TEM-1_* and *bla_OXA_*; *bla_CTX-M_*, *bla_PSE-1_*, *bla_CMY-2_*, and *bla_NDM_* were detected in 5/7 (71.4%) and *bla_SHV_* in 4/7 (57.1%). All isolates carried *stn*, *fimA*, *invA*, *sseL*, and *virK*; *siiE* was present in six of seven isolates. In mice, the biofilm-forming isolate C6469 induced hepatic necrosis and intestinal injury. Microbiome and metabolomic analyses indicated a reduction in beneficial gut bacteria, an increase in pathogenic bacteria, and enhanced phosphatidylcholine (PC) (14:0-P-18:0) metabolism, which in turn promotes liver inflammation. These correlational findings warrant further functional validation. This study highlights food safety implications and the strengthening of surveillance for biofilm-forming *S.* Thompson.

## 1. Introduction

Non-typhoidal *Salmonella* (NTS) is a prevalent zoonotic foodborne pathogen [1] that causes infectious diarrhea and acute gastroenteritis and is ranked among the four leading causes of diarrheal disease globally [2]. It is responsible for approximately 550 million illnesses and nearly 85,000 deaths globally each year [3], and over 70% of foodborne bacterial illnesses in China are caused by NTS through ingestion of contaminated food or water [4]. In recent years, *Salmonella* Thompson, one serotype of NTS, has been known to cause gastroenteritis in humans and animals globally [5,6,7]. The pathogenicity of NTS is predominantly mediated by virulence *genes* encoded on *Salmonella* pathogenicity islands (SPIs), as well as by fimbriae, flagella, and plasmids [8]. One study found that *Salmonella* isolates from dead chick embryos harbored SPI-1 and SPI-2 virulence genes [9]. Accordingly, identifying the virulence *genes* involved in *S.* Thompson pathogenesis is critical for developing infection control strategies.

Antimicrobial treatment currently remains the mainstay of NTS infection control. However, under the selective pressure of antimicrobials, multiple drug resistance (MDR) NTS has become increasingly prevalent [10]. In particular, the extended-spectrum β-lactamases (ESBLs) *bla*_CTX-M_ and *bla_SHV_* have been detected in *S.* Thompson isolated from ready-to-eat pork and chicken products [11,12]. *bla*_CTX-M_ is the most common ESBL type in *Salmonella*, which can disseminate among the family *Enterobacteriaceae* [8]. Biofilm formation has also been observed in S. Thompson in vitro [13]. A biofilm is an extracellular polymeric matrix composed of bacterial-secreted eDNA and extracellular polysaccharides, which exacerbates antimicrobial resistance (AMR) and facilitates its dissemination [14]. When in contact with food products during processing, biofilms may transfer *S.* Thompson to food, causing cross-contamination or recontamination [15]. This not only increases illness and healthcare burdens but also elevates the risk of mortality. One review on AMR estimated that 10 million deaths could be attributable to AMR by 2050 [16]. Clearly, AMR in NTS has emerged as a prevalent issue in public health and food safety globally. Surveillance of AMR in NTS is fundamentally important for preventing its transmission along the food chain [17].

Although *Salmonella* has been detected in a wide range of foods, reports on its presence in edible snails remain scarce. In China, freshwater snails have been reported to harbor *Salmonella* [18]. *Salmonella* has been recovered from edible snails (*Helix spp*.) in Sardinia, including *Salmonella* Arapahoe from a wild-collected snail and *Salmonella* Zanzibar from an imported snail marketed locally [19], and a study in Cameroon detected *Salmonella* spp. among other foodborne pathogens in edible land snails [20]. However, to the best of our knowledge, no study has characterized *S.* Thompson—including its antimicrobial resistance, biofilm formation, virulence *gene* profiles, or pathogenicity—in isolates recovered from *Cipangopaludina cathayensis*, a widely consumed aquatic snail in southern China. This knowledge gap hinders the assessment of the potential public health risk posed by this food reservoir.

In our previous studies, *S.* Thompson was isolated from edible *Cipangopaludina cathayensis* (Gastropoda: Prosobranchia; Mesogastropoda; Viviparidae; *C. cathayensis*) in retail markets. *C. cathayensis* is widely distributed in paddy fields, lakes, marshes, rivers, streams, and ponds in China [21]. Owing to its high edible and medicinal values, with efficacy in alleviating thirst and clearing heat, *C. cathayensis* has become a very important aquatic economic animal in China [22]. In particular, a popular and delicious food named Liuzhou Luosifen, or snail rice noodle, with an annual economic value of 66.99 billion Chinese yuan in 2023 in China, requires the use of *C. cathayensis* [21]. Therefore, the study of *S.* Thompson from *C. cathayensis* is of great significance to public health. *C. cathayensis* is a freshwater snail that inhabits diverse aquatic environments, including paddy fields, lakes, rivers, streams, and ponds, where it feeds on organic matter and microorganisms in the water and sediment. During the preparation of Liuzhou Luosifen, snails are typically subjected to thorough washing and prolonged high-temperature boiling, a process generally sufficient to inactivate vegetative *Salmonella* cells. However, contamination with *S.* Thompson most likely occurs via the fecal–oral route, driven by exposure to contaminated water or sediment during aquaculture, harvesting, transport, or retail handling. Furthermore, the risk of cross-contamination in kitchens where raw snails and ready-to-eat ingredients are handled concurrently, combined with the potential for undercooking, renders *C. cathayensis* a significant vehicle for foodborne *S.* Thompson transmission. Consequently, this issue deserves close attention in risk assessments and hygiene guidelines. However, there is limited information available to evaluate the potential risk factors of *S.* Thompson isolates from edible *C. cathayensis* in southern China. To better understand the characterization of *S.* Thompson originating from edible *C. cathayensis* in two different cities in southern China, namely, Nanning city and Laibin city, this study focused on AMR, biofilms, ESBL- and biofilm-encoding *genes*, and pathogenicity. Our overall aim was to evaluate the potential risk factors of *S.* Thompson to public health and contribute to ensuring food safety in the food chain.

## 2. Materials and Methods

### 2.1. The Sources and Identification of Isolates

A total of 93 edible *C. cathayensis* samples were collected from retail markets in Nanning and Laibin cities, southern China, during 2021–2022. Of these, 36/93 (38.7%) samples were positive for *Salmonella*, yielding multiple serovars. All seven *S.* Thompson isolates recovered from the 36 *Salmonella*-positive samples (C6304, C6458, C6469, C6277, C6465, C6273, and C6272) were selected for further characterization, and no *S.* Thompson isolate was excluded; the remaining non-*S.* Thompson serovars were not included in this study. The isolates were recovered and identified, and the detailed information and recovery procedures are listed in Table S1. During collection, storage, and transport, all samples were handled under aseptic conditions, transported to the laboratory in sterile containers at approximately 4 °C, and processed within 24 h to minimize cross-contamination and alterations in the bacterial flora. All microbiological procedures were performed in a class II biological safety cabinet (Qingdao Haier Biomedical Co., Ltd., Qingdao, China) in a biosafety level 2 (BSL-2) laboratory, and all cultures and contaminated materials were autoclaved prior to disposal. After all samples were collected, bacterial isolation and identification were immediately performed, taking five days to complete each time. A 1 mL aliquot of the isolate cultures was inoculated into 100 mL portions of Nutrient Broth (NB) (Beijing Solarbio Science & Technology Co., Ltd., Beijing, China) and incubated at 37 °C for 18 h, followed by inoculation onto Xylose Lysine Desoxycholate (XLD) agar plates [1]. After a 24 h incubation period at 37 °C, potential *Salmonella* colonies were then individually inoculated into both triple sugar iron and urea agar slants and incubated for an additional 24 h at 37 °C. The isolate serotypes were determined by the slide agglutination test using the Kauffman–White serotyping method with anti-*Salmonella* serum.

### 2.2. Antimicrobial Susceptibility Testing

Antimicrobial susceptibility testing was carried out on 23 May 2023 and completed on 30 May 2023. According to CLSI Guidelines [23], antimicrobial susceptibility of *S.* Thompson isolates was determined using the Kirby–Bauer disk diffusion method. For each strain, a fresh culture was adjusted to the logarithmic growth phase (approximately 1 × 10^8^ CFU/mL, equivalent to a 0.5 McFarland standard) and spread onto Mueller–Hinton agar plates. A total of 30 antimicrobial agents were applied (Hangzhou Microbial Reagent Co., Ltd., Hangzhou, China). *Escherichia coli* (ATCC 25922; Manassas, VA, USA) was used as a quality control organism. The logarithmic bacterial suspension was spread onto a sterile agar plate, and then an antimicrobial disk was placed on top. The plate was inverted and cultured at 37 °C for 24 h, after which the diameter of the inhibition zone was measured. A complete list of the disk contents and interpretive criteria (zone diameter and MIC breakpoints) applied to all 30 agents is provided in Table S5. Among the 30 agents, ten agents lacked valid CLSI interpretive criteria for non-typhoidal *Salmonella* and were therefore reported descriptively and excluded from the MDR classification: vancomycin, clindamycin, lincomycin, erythromycin, oxacillin, and penicillin (no criteria established for Enterobacteriaceae); florfenicol (no criteria in M100); polymyxin B (disk diffusion criteria not established; broth microdilution only); levofloxacin (no disk diffusion criteria for *Salmonella*; MIC criteria only); and azithromycin (criteria apply only to *Salmonella* Typhi). The per-isolate results are provided as a reclassified antimicrobial resistance matrix in Table S4. An MDR strain is defined as an isolate demonstrating resistance to three or more antimicrobials in different antimicrobial classes [8].

### 2.3. Screening for Biofilm-Forming Strains

A crystal violet staining method (Beijing Solarbio Science & Technology Co., Ltd., Beijing, China) was used to identify the strains with biofilm formation ability [14]. The detection of biofilms was completed in November 2023, taking 40 days. This duration reflects the total time required to complete the assays for all isolates and replicates and does not represent the incubation time of a single assay. For each isolate, the assay was performed in three biological replicates, each with three technical replicates; sterile uninoculated broth was used as the negative control (ODc); absorbance was measured using a microplate reader (Tecan Spark, INFINITE 200 PRO; Tecan Group Ltd., Männedorf, Switzerland). Detailed operating method: The activated bacterial solution was inoculated into 96-well microtiter plates (Shandong Sikejie Biotechnology Co., Ltd., Jinan, China) to a final concentration of 1 × 106 CFU/mL and then incubated for 48 h until the biofilm attained maturity. At first, most of the bacterial solution was decanted and washed twice with phosphate-buffered saline (PBS) (BioMedia Corp, Singapore) to remove any non-adherent cells. Subsequently, methanol (Chengdu Kelong Chemical Co., Ltd., Chengdu, China) was added for fixation for 15 min, after which the methanol was discarded and allowed to evaporate completely. Then, crystal violet staining was performed for 5 min, after which the crystal violet was discarded. After being washed with distilled water until the effluent was colorless, anhydrous ethanol (Chengdu Kelong Chemical Co., Ltd., Chengdu, China) was added for decolorization. Finally, absorbance was measured at 570 nm by a microplate reader. The procedures of the crystal violet staining method and the classification criteria for biofilm formation ability are listed in Table S2. This threshold-based classification, together with three biological replicates (each with three technical replicates) per isolate and the use of sterile uninoculated broth as a negative control, provided the basis for discriminating biofilm-positive from biofilm-negative strains. A dedicated positive control strain was not included in the biofilm assay; this is acknowledged as a methodological limitation of this study.

### 2.4. Detection of β-Lactamase-Encoding Genes, Biofilm-Encoding Genes and Virulence Genes

Seven *S.* Thompson isolates were screened for biofilm-encoding *genes*, virulence *genes*, and β-lactamase-encoding *genes* by polymerase chain reaction (PCR) (Thermo Fisher Scientific, Waltham, MA, USA). The PCR experiment was completed in April 2024, taking 45 days. All primers were obtained from previously published studies. Their specificity was validated using previously confirmed *Salmonella* isolates and reference strains as positive and negative controls, respectively, and the expected amplicon sizes were confirmed for all targets. All *gene* primer information and the conditions of the PCR are listed in Table S3. All the PCR products were directly sequenced by BGI Co., Ltd. (Beijing, China) to confirm the accuracy of target *genes* of PCR [8,14].

### 2.5. Pathogenicity of Isolates

The biofilm-forming isolate C6469, which harbored the complete set of tested β-lactamase *genes* and the biofilm-associated *genes csgA* and *csgD* and exhibited the strongest biofilm-forming capacity, was selected for the in vivo experiment because it was considered representative of the most concerning phenotype among the isolates. Specific Pathogen-Free (SPF) Kunming species of mice (Hunan SJA Laboratory Animal Co., Ltd., Changsha, China), weighing between 18 and 22 g, with an equal number of males and females, were used for a week of adaptive housing with unrestricted access to water and food. This experiment adhered to the principles of animal welfare and was approved by the Guangxi University of Chinese Medicine Institutional Welfare and Ethical Committee (Approval No. DW20230830-170). During our experiments, we made every effort to control confounding factors. For example, the order of animal experiments was maintained consistently each time and conducted by the same person.

A formal a priori power or sample size calculation was not performed; the group size (*n* = 10 per group) was instead chosen as the minimum number of animals considered necessary to detect the expected effects in this infection model while adhering to animal welfare principles. This is acknowledged as a limitation of this study. Twenty mice were randomly divided equally into two groups: a control group and an infection group. A completely random method was used to group the animals. The control group received sterile physiological saline (0.2 mL/10 g body weight) via oral gavage, while the infection group received an equal volume of *S.* Thompson (C6469, 1 × 10^8^ CFU/mL) suspension, administered twice. The mice were monitored daily for clinical signs (including activity, fur condition, appetite, and body weight) as well as morbidity and mortality throughout the 7-day observation period. Seven days later, in accordance with the guidelines set forth by the American Veterinary Medical Association, the mice were anesthetized using 2% isoflurane (Nanjing Aibei Biotechnology Co., Ltd., Nanjing, China; Specification: M2910) inhalation and weighed, after which blood was drawn. The euthanasia procedure was then carried out by inducing overexposure to the anesthetic isoflurane. Organs were then harvested and weighed to determine the organ index, identify pathological changes and evaluate the presence of *S.* Thompson [24]. In addition, blood non-targeted metabolomics and intestinal content quantitative microbiome analyses were performed by Shanghai G&C biotech Co., Ltd. (Shanghai, China). Animal experiments were evaluated according to the ARRIVE checklist, and the information is available at https://www.scidb.cn/en/anonymous/bk1CQkp2 [25] (accessed on 10 October 2025). The pathogenic experiment started on 1 September 2023 and was completed on 10 July 2024.

### 2.6. PFGE Analysis

Pulsed-field gel electrophoresis (PFGE) was performed to determine the genetic relatedness of the seven isolates. The PFGE experiment was completed in October 2024. Genomic DNA embedded in agarose plugs was digested with the restriction enzyme XbaI, and the resulting fragments were separated following the standardized PulseNet protocol. The electrophoresis conditions were as follows: initial switch time 2.2 s, final switch time 54.2 s, run time 20 h, angle 120°, gradient 6 V/cm, and temperature 4 °C. Gels were stained with ethidium bromide (0.5 mg/mL for 30 min), and the DNA fingerprints were visualized under UV illumination (Uvitec, Lodi, NJ, USA). PFGE profiles were compared using the Dice similarity coefficient, and cluster analysis was performed by the unweighted pair-group method with arithmetic mean (UPGMA), with an 80% similarity cutoff to define clonal groups [17].

### 2.7. Statistical Analysis

Statistical analyses were performed using GraphPad Prism version 8.0 (GraphPad Software, San Diego, CA, USA) and R software (version 4.4.2). One-way analysis of variance was used for parametric comparisons after verifying the homogeneity of variances; when this assumption was violated, the non-parametric Kruskal–Wallis test was applied. A *p*-value < 0.05 was considered statistically significant.

For gut microbiota analyses, α-diversity indices—including observed species, Chao1, ACE, Shannon, and Simpson—were compared between groups using the Kruskal–Wallis test. β-Diversity was assessed by principal coordinate analysis (PCoA) based on Bray–Curtis dissimilarity, and the significance of intergroup separation was evaluated using permutational multivariate analysis of variance (PERMANOVA; Adonis, 999 permutations). Linear discriminant analysis effect size (LEfSe) was employed to identify taxa with significantly different abundances between groups (LDA score > 4.0, *p* < 0.05).

For metabolomics analyses, differentially abundant metabolites were identified using orthogonal partial least-squares discriminant analysis (OPLS-DA) combined with variable importance in projection and the Wilcoxon rank-sum test (*p* < 0.05). KEGG pathway enrichment analysis (https://www.bioinformatics.com.cn) was performed using the hypergeometric test (*p* < 0.05).

Spearman’s rank correlation analysis was used to assess correlations between metabolites and gut microbiota genera, as well as between biofilm-forming ability and the presence of resistance or virulence *genes*. The resulting correlation network was visualized using Cytoscape (version 3.7.2), and the significance of network edges was tested (*p* < 0.05). Multiple testing corrections were applied using the Benjamini–Hochberg false discovery rate (FDR) method where appropriate.

### 2.8. Study Period

The experimental period began with the collection of snail samples on 9 February 2021 and ended upon the completion of the last PFGE experiment on 29 October 2024.

## 3. Results

### 3.1. MDR Was Commonly Observed in Isolates

The isolates showed different degrees of reduced susceptibility to most of the 30 tested antimicrobials, with the exception of polymyxin B (PB), imipenem (IPM), amikacin (AMK), gentamicin (GEN), and kanamycin (KAN) (Figure 1A, Table 1). In particular, the resistance rates of antimicrobials, including lincomycin (MY), vancomycin (VAN), clindamycin (CC), erythromycin (E) and oxacillin (OX), were 100%. However, because no CLSI interpretive criteria are available for these agents in *Salmonella*, these data are reported descriptively and were excluded from the MDR classification. After this reclassification, two isolates, 2/7 (28.6%; C6277 and C6465), met the MDR definition (resistance to three or more antimicrobial classes with valid interpretive criteria), whereas the other five isolates were resistant to at most one antimicrobial class with valid criteria (Figure 1B). In particular, one strain (C6465) was resistant to 24 of the tested antimicrobials. Another notable phenomenon was that all isolates had reduced antimicrobial susceptibility to PEN, CN, CZ and CPZ.

### 3.2. Biofilm Formation of Isolates Was Associated with Harboring Biofilm-Encoding Genes

Strong biofilm formation abilities of five isolates were observed (Figure 1C). Consistently, the biofilm-encoding *genes csgA* or *csgD* were harbored by the five isolates that were capable of biofilm formation. In contrast, strains (C6273, C6272) that did not form biofilms did not harbor those *genes* (Figure 2). Spearman correlation analysis revealed a statistically significant association between the presence of *csgA/csgD* and biofilm-forming ability (*p* < 0.05); however, given the limited sample size, this finding should be interpreted as correlational rather than as evidence of a causal relationship.

### 3.3. Prevalence of β-Lactamase-Encoding Genes and Virulence Genes

All the isolates harbored at least two β-lactamase-encoding *genes,* including *bla_TEM-1_* and *bla_OXA_* (Figure 2). Overall, 5/7 (71.43%) of the isolates were positive for *bla_CTX-M_, bla_PSE-1_*, *bla_CMY-2_*, and *bla_NDM_*, followed by 4/7 (57.14%) that harbored *bla_SHV_.* Notably, *bla*_CTX-M_, *bla*_SHV_*,* and *bla_TEM_* encode ESBLs; *bla_CMY_*_-2_ encodes an AmpC β-lactamase; and *bla_NDM_* encodes a carbapenemase. Accordingly, these *genes* are reported and interpreted separately throughout this manuscript. Four isolates (57.14%) also harbored all seven β-lactamase *genes*. A reliable gel interpretation condition for the *bla_NDM_* amplicon could not be established during assay optimization; therefore, the *bla_NDM_* result should be regarded as provisional. All the isolates harbored at least five of eight virulence *genes*, including *stn*, *fimA*, *virK*, *invA* and *sseL*. The *gene siiE* was not detected in one *S.* Thompson isolate (C6273), and the *sopB gene* was not detected in two isolates (C6458, C6469). In addition, the *mgtC gene* was not detected in two isolates (C6465, C6469). Notably, all *S.* Thompson isolates harbored the virulence *genes* and β-lactamase-encoding *genes*.

Notably, the virulence *gene* profiles (C6273, C6272 or C6465, C6469) and β-lactamase types (C6469, C6458) of strains from the same city were different, while those of strains (C6273, C6458) from different cities were unexpectedly consistent.

### 3.4. PFGE Analysis Revealed Four Genetic Patterns Among the Isolates

Four PFGE patterns were identified among the seven *S.* Thompson isolates (Figure 2). Closely related PFGE profiles (Dice similarity > 80%) were observed among isolates C6304, C6458, and C6469, which were recovered from two different cities (Nanning and Laibin), suggesting possible cross-transmission among aquatic animals in these regions. Isolates C6277 and C6465 also shared closely related PFGE profiles. Overall, isolates from Laibin city showed the highest genetic diversity (four PFGE patterns), followed by those from Nanning city (two PFGE patterns). Notably, isolates C6304, C6458, and C6469 not only shared closely related PFGE profiles but also exhibited highly similar antimicrobial resistance and biofilm formation phenotypes. These results are consistent with clonal relatedness among some isolates; however, given the limited number of isolates and the absence of whole-genome-sequencing-based confirmation, definitive conclusions on clonal relatedness and transmission routes cannot be drawn.

### 3.5. The Livers and Intestines of Mice Were Damaged by Isolates

Multiple necrotic spots were observed on the livers of the challenged Kunming mice (Figure 3A). Unexpectedly, the spleen index of the infected mice did not significantly increase, whereas the liver index decreased significantly (Figure 3B,C). *S.* Thompson was detected in both liver and intestinal tissues, with significantly higher levels than in the blank group (Figure 3D,E). The histopathology results showed obvious vacuoles in the cytoplasm or nucleus of hepatocytes, with the whole cell exhibiting a honeycomb structure (Figure 4), and the intestinal villi and glands were damaged (Figure 5). No mortality was observed in any group throughout the 7-day observation period. Because only one isolate (C6469) was tested in the animal model, these in vivo data demonstrate the high pathogenicity of this particular isolate in mice and cannot be extrapolated to the remaining six isolates.

### 3.6. The Abundance of Gut Microbiota Was Destroyed by S. Thompson Isolates

Compared with the blank group, the model group showed a statistically insignificant decrease in observed species, while the Chao1, ACE, Shannon, and Simpson indices remained stable (*p* > 0.05). This indicated that *Salmonella* infection caused no significant changes in the actual microbial community species count. All sequencing coverage values exceeded 0.99, demonstrating sufficient sequencing depth and reliable results. β-Diversity was analyzed through principal component analysis (PCA), principal coordinate analysis (PCoA), and non-metric Multidimensional Scaling (NMDS). The confidence ellipse of the model group could be separated from that of the blank group (*p* < 0.05), indicating that *Salmonella* infection significantly altered the overall structure of the gut microbiota. At the genus level of the gut microbiota, Lactococcus, the Eubacterium oxidoreducens group, Herminiimonas, and others exhibited significant increases (*p* < 0.05) (Figure 6A). At the species level of the gut microbiota, Streptococcus_danieliae showed significant increases (*p* < 0.05), whereas [Clostridium]_leptum and Adlercreutzia_mucosicola decreased (*p* < 0.05) (Figure 6B). Based on LEfSe clustering analysis, the model group was dominated by g__Jeotgalicoccus, g__Mammaliicoccus, s__Streptococcus danieliae and o__Flavobacteriales (LDA > 6.0) (Figure 6C).

### 3.7. Intestinal–Liver Inflammation Was Caused by S. Thompson via the Intestinal Flora–Metabolite–Liver Pathway

The heat map shows the relative content of all significantly different metabolites in different samples. The intergroup difference is greater than the intragroup difference, and the experimental group effect is highly significant and reliable. The metabolite PC (14:0-P-18:0) was significantly upregulated (*p* < 0.05), and Isoorientin and L-Lysopine were significantly downregulated (*p* < 0.05). Most notably, the tricarboxylic acid cycle, along with amino acid metabolic pathways, such as tryptophan metabolism, phenylalanine metabolism, and pyruvate metabolism, were inhibited. Taurine and hypotaurine metabolism were among the few pathways that were significantly activated (Figure 7A). Metabolite–gut microbiota analysis revealed strong positive correlations between Streptococcus, Jeotgalicoccus, Muribaculaceae_genus, Mammaliicoccus, and Flavobac-teriaceae_genus with metabolites such as PC (14:0-P-18:0) and UDP-N-acetyl-D-glucosaminuronate (Figure 7B). Conversely, Escherich-ia–Shigella and Paraprevotella exhibited significant negative correlations with these metabolites. This suggests that different bacterial communities may collectively regulate key metabolite levels through either synergistic or antagonistic interactions. PC (14:0-P-18:0) has established connections with multiple bacterial genera. Notably, the relationship between PC (14:0-P-18:0) and Angelakisella exhibits a significant linear negative correlation trend. Additionally, we identified several interesting correlations: the strongest negative specific correlation was observed between PC (14:0-P-18:0) and Roseburia; the strongest negative specific correlation was observed between Roseburia and L-phenylalanine; and the strongest negative specific correlation was observed between L-phenylalanine and Adlercreutzia. A total of 77 bacterial genera and metabolite-related nodes were identified within the entire data network, along with 106 significantly correlated edges (Figure 7C). As these analyses are correlational in nature, the proposed “gut microbiota–metabolite–liver” pathway is presented as a hypothesis that warrants further functional validation.

## 4. Discussion

*S.* Thompson is a foodborne pathogen that can infect humans and cause gastroenteritis. It has a wide range of hosts, including chickens and their products, beef, cilantro, rocket lettuce, eggs, milk, and fish [1,5,26]. However, no information is available on *S.* Thompson isolated from *C. cathayensis*. This study is the first to report the potential threat to public health posed by *S.* Thompson isolated from edible *C. cathayensis* in China. The present study did not evaluate the implementation or effectiveness of HACCP systems; the findings nevertheless support recommendations for strengthened *Salmonella* monitoring and food safety control measures in aquatic animals. In this study, strains from the same city had different virulence *gene* profiles and β-lactamase types, while those of strains from different cities were unexpectedly consistent. These results indicate possible genetic relatedness among *S.* Thompson from different cities or the same city, suggesting that transmission occurred among aquatic animals within these regions. PFGE analysis identified four distinct profiles and revealed closely related profiles (Dice similarity > 80%) among three isolates from the two cities (C6304, C6458, and C6469), which is consistent with possible clonal relatedness and cross-transmission among aquatic animals in Nanning and Laibin, although whole-genome-sequencing-based confirmation is required. However, given the limited number of isolates, these findings should be regarded as preliminary and warrant confirmation by whole-genome sequencing in future studies. Once contaminated food is consumed by humans, it poses a significant public health threat. Therefore, it is of great significance to strengthen *Salmonella* surveillance in aquatic animals to reduce the threat to public health.

The greatest threat to public health posed by *Salmonella* stems from its pathogenicity [27]. Research has demonstrated that *S.* Thompson isolated from the bloodstream of infected patients and from patients with gastroenteritis exhibits high pathogenicity [7,28]. This study also showed that *S.* Thompson could induce liver necrosis and damage hepatocytes, intestinal villi, and glands, exhibiting high pathogenic characteristics and indicating a significant public health threat. The pathogenicity of *Salmonella* is primarily linked to its virulence *genes*. In our previous study, we found that high *Salmonella* virulence was associated with the SPI-2 *gene sseL*, as the protein encoded by this *gene* protects *Salmonella* from macrophages [24]. Interestingly, *S.* Thompson was also found to carry the *sseL gene*, suggesting this gene may be responsible for its pathogenic potential. In addition to the *sseL gene*, *S.* Thompson harbors six other virulence *genes*, all of which contribute to increased virulence. The *mgtC gene* encodes proteins that promote bacterial survival within macrophages, while the *sopB gene* encodes proteins that could promote inflammatory responses within the intestinal mucosa [29,30]. Interestingly, in vivo pathogenicity of S. Thompson (C6469) was observed despite the absence of the *sopB* and *mgtC genes*; whether other factors functionally compensate for this deficiency remains untested and requires direct experimental validation.

In addition to virulence *genes*, the disruption of intestinal flora homeostasis may be another potentially more important pathogenic mechanism [31]. In this study, beneficial bacteria, including Adlercreutzia, Roseburia, and Angelakisella, declined after *Salmonella* infection. Conversely, harmful bacteria, such as Streptococcus and the genus Flav Bacteriaceae, increased. This suggests that *Salmonella* infection disrupts the intestinal flora balance in mice and promotes the metabolism of pathogenic bacteria, thereby facilitating their infectious role. Furthermore, intestinal pathological sections confirmed that following *Salmonella* infection, the intestinal villi and mucosa were destroyed. These pathological changes suggest that *Salmonella* infection increases intestinal mucosal permeability and damages the mucosal barrier. Blood metabolome analysis further supported this hypothesis. The abundance of metabolites in the blood exhibited a significant correlation with alterations in intestinal bacterial genera, suggesting that gut microbiota metabolites enter the bloodstream due to heightened intestinal mucosal permeability or mucosal barrier impairment [32]. In this study, PC (14:0-P-18:0) attracted particular attention. Its levels not only significantly increased following *Salmonella* infection but also established both synergistic and antagonistic interactions with multiple bacterial genera. These findings indicate that changes in its concentration are closely associated with alterations in the overall structure of the gut microbiota. PC (14:0-P-18:0) (1-methylnonanoxy-2-acetoheptano-18:0), a specific acetaldehyde phospholipid, plays a complex and crucial role in regulating inflammatory responses and cellular survival through its metabolites. PC (14:0-P-18:0) participates in phospholipase-mediated signaling pathways. When hydrolyzed by phospholipase A_2_ (PLA_2_), it releases the fatty acid at the sn-2 position (i.e., acetaldehydeated nonanoic acid P-18:0), generating lyso-phosphatidylcholine (1-stearoyl-2-lyso-sn-glyco-3-PC, Lyso-PC) and a free fatty acid [33]. High concentrations of Lyso-PC are a “danger signal”. It activates G protein-coupled receptors, including G2A and GPR4, on various immune cells, such as macrophages and T cells, resulting in the secretion of pro-inflammatory cytokines, including TNF-α, IL-1β, and IL-6, among others [34,35]. This is commonly observed in pathological conditions, such as tissue injury and acute inflammation, where a significant number of cells die and phospholipase A2 (PLA2) is activated, resulting in the production of large amounts of Lyso-PC. We therefore speculate that *Salmonella* infection can activate the host cell’s lipid metabolism, particularly phospholipase metabolism, leading to intestinal and liver inflammation. Furthermore, species of Roseburia, which exhibited a negative correlation with PC (14:0-P-18:0), also demonstrated a negative correlation with L-phenylalanine. Following *Salmonella* infection, the phenylalanine metabolic pathway was suppressed, leading to an inability to metabolize phenylalanine in the serum and an increase in its concentration. However, elevated levels of phenylalanine can impair mitochondrial function, provoke oxidative stress and inflammatory responses, and may even lead to cell apoptosis. This could contribute to neurological dysfunction and liver damage [36]. Therefore, following *Salmonella* infection, the activity of beneficial bacteria within the intestinal flora is suppressed, which diminishes the host’s ability to utilize phenylalanine, leading to liver inflammation and damage.

Additionally, one study observed that the virulence of bacteria could be enhanced by their high resistance [37]. In this study, two isolates exhibited an MDR phenotype, which may be associated with enhanced virulence, and all isolates harbored multiple β-lactamase *genes*, especially TEM and OXA. To our knowledge, this is the first report in which bla_OXA_ and bla_TEM-1_ were detected in *S.* Thompson strains in China. TEM is a class A enzyme of β-lactamase, and OXA is a class D serine carbapenemase, which confers bacterial resistance to β-lactam antimicrobials [38]. This suggests that *S.* Thompson isolated from *C. cathayensis* may develop resistance to more β-lactam antimicrobials in the future. The spread of antimicrobial resistance *genes* remains an important public health concern [39]. The common ESBL type in Salmonella in China is the CTX-M group, which is usually located on transmissible plasmids and can spread among Enterobacteriaceae [40]. In this study, isolates exhibiting strong genetic relatedness and originating from various sources were also found to carry CTX-M, indicating that it may have been transmitted among these strains. Consequently, continuous surveillance of Salmonella in aquatic animals is crucial for food safety control. However, in the absence of molecular typing data, the genetic relatedness among these strains remains unresolved, and this study does not provide direct evidence of plasmid-mediated transmission. Some strains remained phenotypically susceptible to β-lactam antimicrobials despite carrying ESBL-encoding *genes*. This genotype–phenotype discrepancy may reflect the lack or low-level expression of these *genes*, regulatory effects, non-functional *gene* variants, substrate specificity of the encoded enzymes, or methodological limitations of phenotypic testing; *gene* detection alone should not be interpreted as evidence of function. Notably, *S.* Thompson isolates formed biofilms in vitro. One study found that biofilm formation in *Salmonella* increased AMR by up to 1000-fold [14] and enhanced virulence by 20% [37]. Notably, *S.* Thompson (C6469), which lacks *sopB* and *mgtC*, exhibited significant biofilm-forming ability; the coexistence of biofilm-forming capacity, resistance determinants, and virulence-associated characteristics is reported here, whereas any causal or compensatory relationship among them remains untested and requires direct experimental validation (e.g., *gene* knockout or complementation studies). Thus, biofilm formation may represent a contributing factor to both pathogenic phenotypes and MDR in *S.* Thompson. Moreover, *S.* Thompson isolates that lack the *csgA* and *csgD genes* cannot form biofilms in vitro, which is consistent with previous studies [41,42]. Biofilm formation is associated with specific *genes*. The pilus protein CsgD, encoded by *csgD*, facilitates biofilm formation by transcriptionally activating the *csgBAC* operon, thereby enhancing bacterial adhesion [43]. Consequently, to mitigate the public health risks posed by *Salmonella*, it is necessary to monitor not only antimicrobial resistance (AMR) and pathogenicity but also biofilm formation.

This study has several limitations. First, only seven *S.* Thompson isolates recovered from 36 *Salmonella*-positive samples were characterized, and the other *Salmonella* serovars recovered from the same samples were not included. Although the total number of samples (*n* = 93) and the number of *Salmonella*-positive samples (*n* = 36) were reported, the small number of *S.* Thompson isolates (*n* = 7) limits the statistical power and the generalizability of the epidemiological inference. Second, sampling was restricted to two cities in southern China, and the sampling times spanned 2021–2022 without perfect matching between the two cities. Third, only one biofilm-forming isolate (C6469) was tested in the animal model, so the in vivo findings cannot be extrapolated to all isolates. Fourth, the *bla_NDM_* result should be regarded as provisional because a reliable gel interpretation condition could not be established. Fifth, genetic relatedness was assessed by PFGE only, and whole-genome-sequencing-based typing, plasmid analysis, and *gene* expression studies were not performed. Finally, the microbiota–metabolite analyses were correlational, and the proposed “gut microbiota–metabolite–liver” pathway requires functional validation.

## 5. Conclusions

This study demonstrated that antimicrobial resistance was prevalent among *S.* Thompson isolates from edible *C. cathayensis*. All isolates, 7/7 (100%), exhibited resistance to at least one antimicrobial agent with valid interpretive criteria; two isolates, 2/7 (28.6%), met the MDR definition, whereas the remaining five isolates were resistant to at most one antimicrobial class with valid interpretive criteria. Five isolates, 5/7 (71.4%), were biofilm producers carrying the biofilm-associated *genes csgA* and *csgD*, whereas the non-biofilm-forming isolates lacked these *genes*. Furthermore, all isolates, 7/7 (100%), harbored multiple β-lactamase- and virulence-encoding *genes*. In a mouse model, infection with the biofilm-forming isolate C6469 reduced the abundance of beneficial intestinal bacteria, promoted phospholipid-related metabolism, and induced hepatic inflammation, suggesting a potential pathogenic mechanism that warrants further functional validation. Given the small sample size (*n* = 7), limited geographic coverage, and correlational nature of the microbiota–metabolite analyses, the proposed “gut microbiota–metabolite–liver” axis should be regarded as a hypothesis. These findings underscore the need for enhanced surveillance of *Salmonella* in aquatic animals and for further investigations with larger sample sizes, including molecular typing, to fully assess the potential public health threat posed by *S.* Thompson in edible *C. cathayensis*.

## Figures and Tables

**Figure 1:**
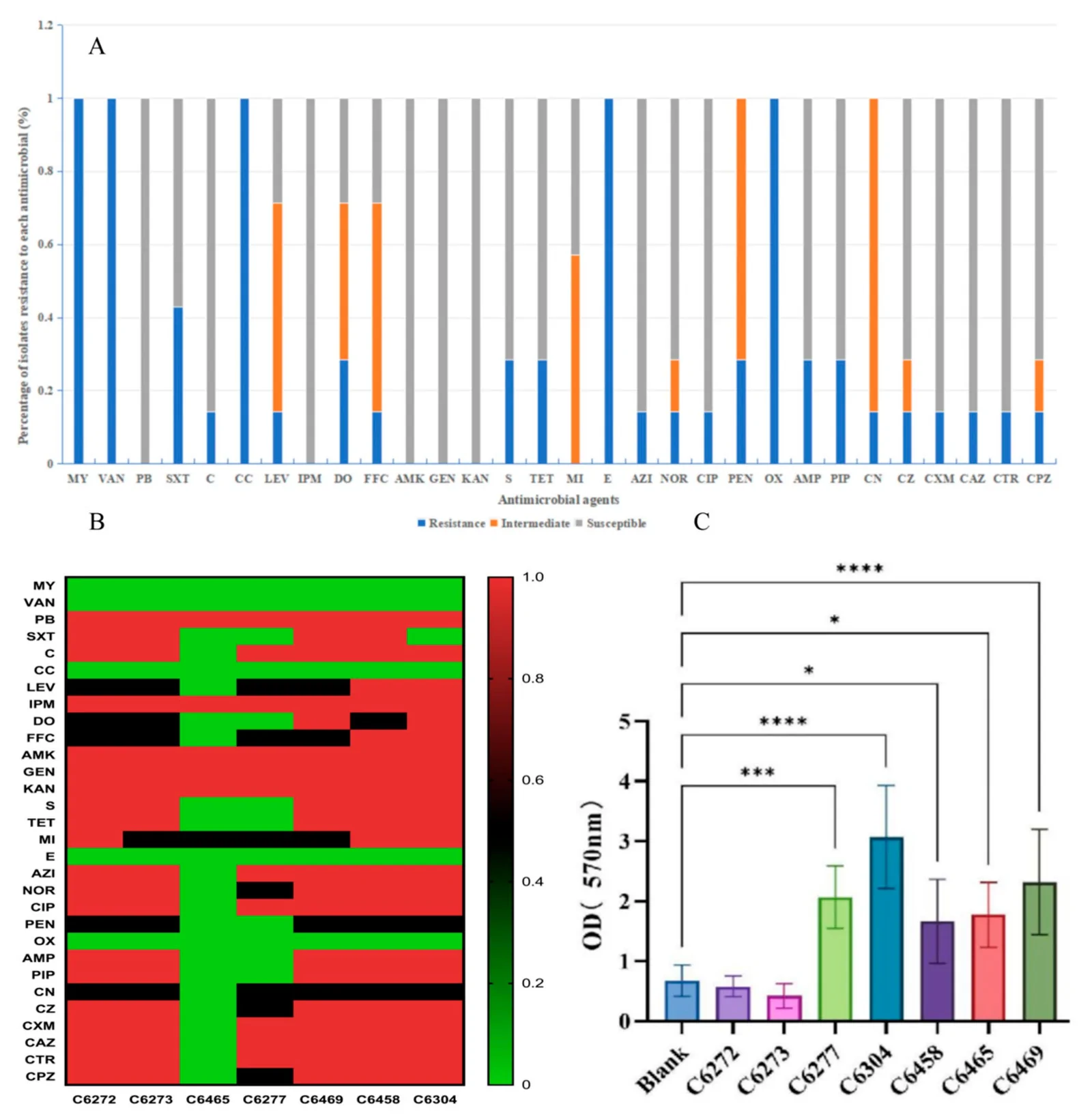
Characterization of resistance in *S.* Thompson isolates. (**A**) Percentage of isolates with resistance to each antimicrobial (%). (**B**) The number of AMR. (**C**) The capacity for biofilm formation. Lincomycin (MY), vancomycin (VAN), polymyxin B (PB), trimethoprim/sulfamethoxazole (SXT), chloramphenicol (C), clindamycin (CC), levofloxacin (LEV), imipenem (IPM), doxycycline (DO), florfenicol (FFC), amikacin (AMK), gentamicin (GEN), kanamycin (KAN), streptomycin (S), tetracycline (TET), minocycline (MI), erythromycin (E), azithromycin (AZI), norfloxacin (NOR), ciprofloxacin (CIP), penicillin (PEN), oxacillin (OX), Ampicillin (AMP), piperacillin (PIP), cephalexin (CN), cefazolin (CZ), cefuroxime sodium (CXM), ceftazidime (CAZ), ceftriaxone (CTR), and cefoperazone (CPZ). * indicates *p* < 0.05, *** indicates *p* < 0.001, and **** indicates *p* < 0.0001.

**Figure 2:**
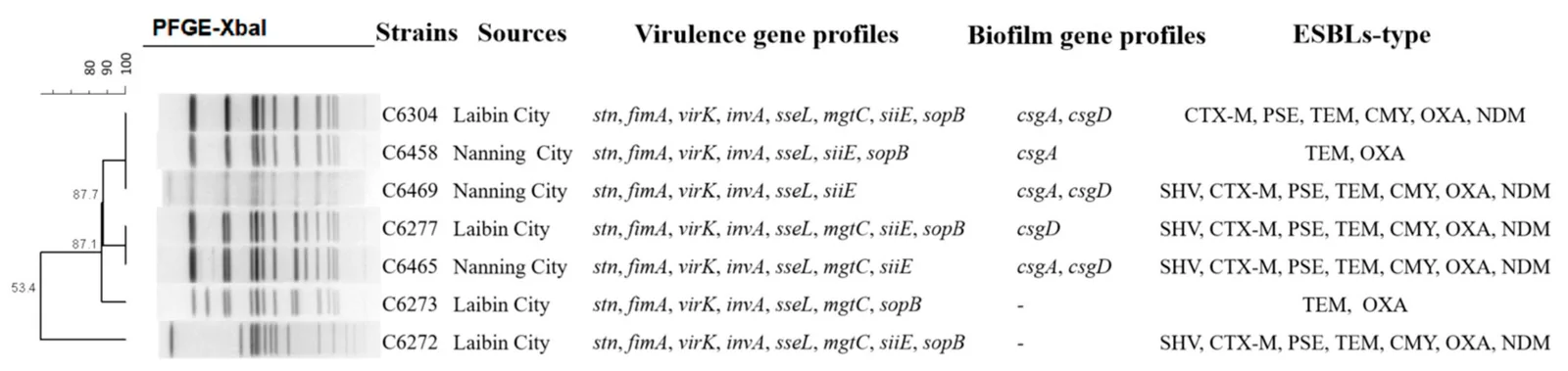
PFGE–XbaI analysis of the seven *S.* Thompson isolates. The dendrogram was generated by UPGMA cluster analysis based on the Dice similarity coefficient (cutoff 80%); the right panel summarizes the source city, virulence *gene* profile, and biofilm *gene* profile.

**Figure 3:**
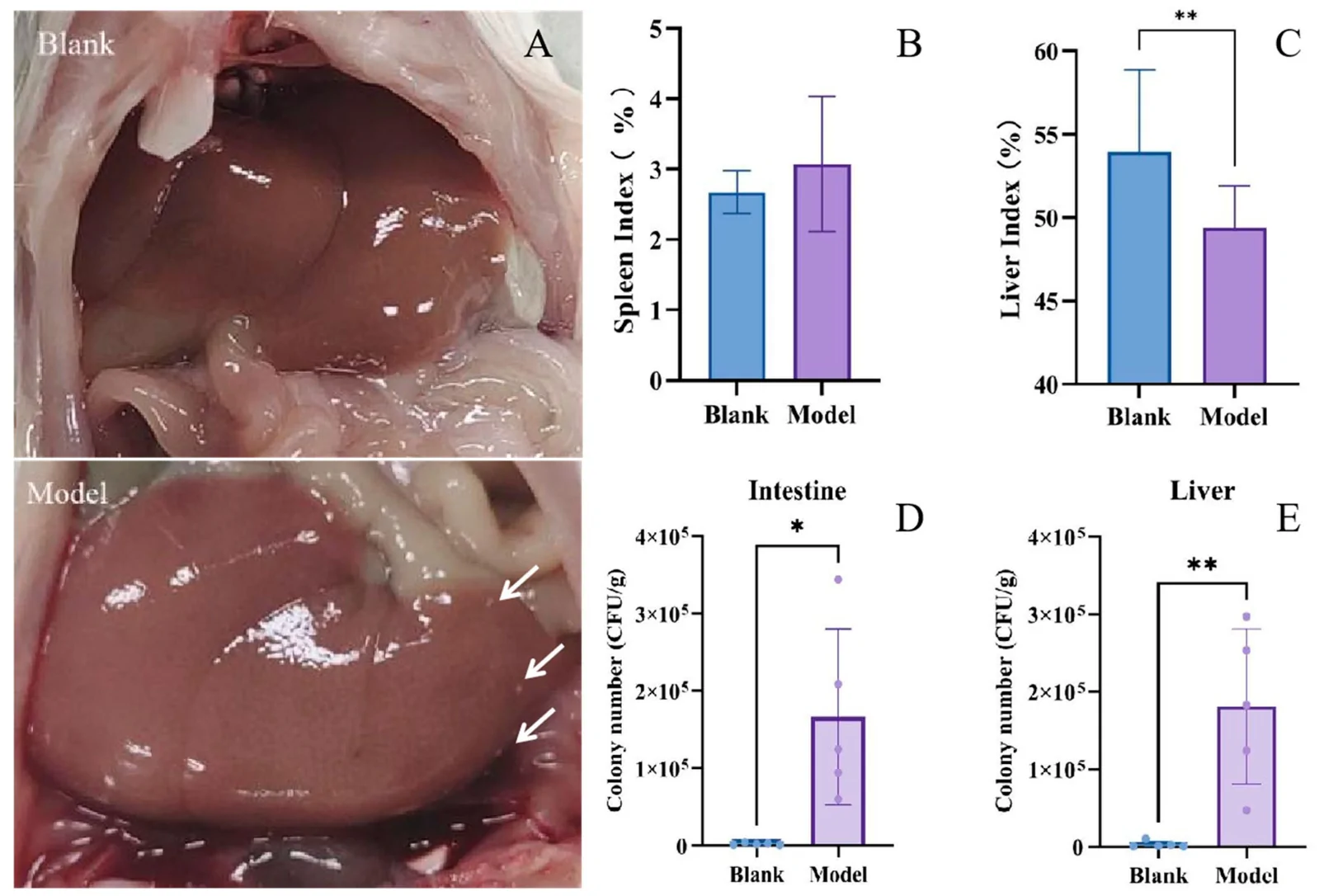
Pathogenicity of *S.* Thompson isolates. (**A**) Liver lesions of the challenged Kunming mice. White arrows indicate necrotic spots. (**B**) Spleen index. (**C**) Liver index. (**D**) Colony number in the intestine. (**E**) Colony number in the liver. The liver index is an indicator used to measure the relative size of the liver. The calculation method is usually: liver index = liver weight (g)/animal body weight (g) × 100%. The spleen index is an indicator to measure the spleen. The calculation method is usually: spleen index = spleen weight (g)/body weight (g) × 100%. *: * (*p* < 0.05), ** (*p* < 0.01).

**Figure 4:**
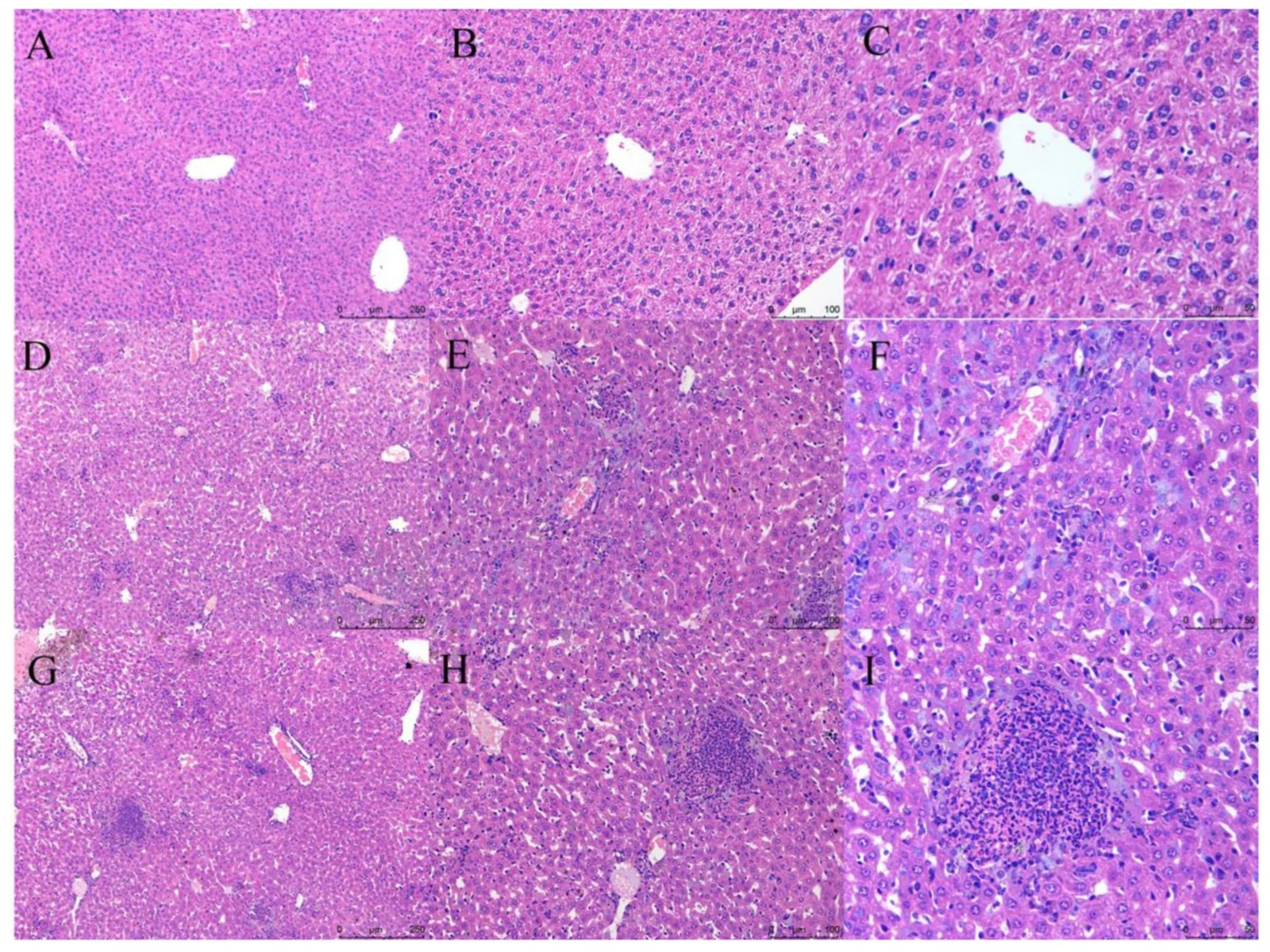
Liver pathology after *S.* Thompson infection. (**A**) Blank group, ×100. (**B**) Blank group, ×200. (**C**) Blank group, ×400. (**D**) Infection group with a concentration of 1 × 10^8^ CFU/mL *S.* Thompson, ×100. (**E**) Infection group with a concentration of 1 × 10^8^ CFU/mL *S.* Thompson, ×200. (**F**) Infection group with a concentration of 1 × 10^8^ CFU/mL *S.* Thompson, ×400. (**G**) Infection group with a concentration of 1 × 10^9^ CFU/mL *S.* Thompson, ×100. (**H**) Infection group with a concentration of 1 × 10^9^ CFU/mL *S.* Thompson, ×200. (**I**) Infection group with a concentration of 1 × 10^9^ CFU/mL *S.* Thompson, ×400.

**Figure 5:**
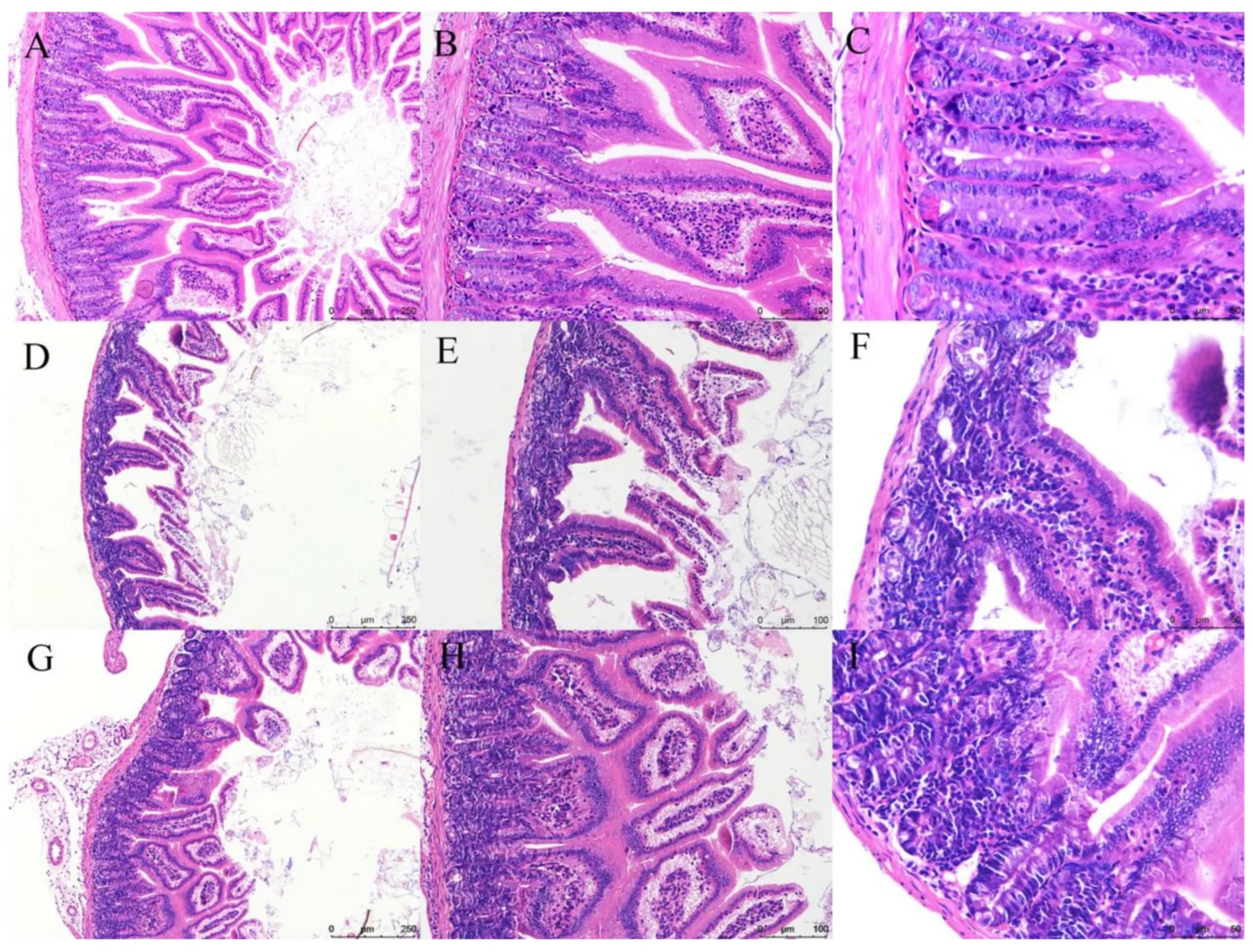
Intestinal pathology after *S.* Thompson infection. (**A**) Blank group, ×100. (**B**) Blank group, ×200. (**C**) Blank group, ×400. (**D**) Infection group with a concentration of 1 × 10^8^ CFU/mL *S.* Thompson, ×100. (**E**) Infection group with a concentration of 1 × 10^8^ CFU/mL *S.* Thompson, ×200. (**F**) Infection group with a concentration of 1 × 10^8^ CFU/mL *S.* Thompson, ×400. (**G**) Infection group with a concentration of 1 × 10^9^ CFU/mL *S.* Thompson, ×100. (**H**) Infection group with a concentration of 1 × 10^9^ CFU/mL *S.* Thompson, ×200. (**I**) Infection group with a concentration of 1 × 10^9^ CFU/mL *S.* Thompson, ×400.

**Figure 6:**
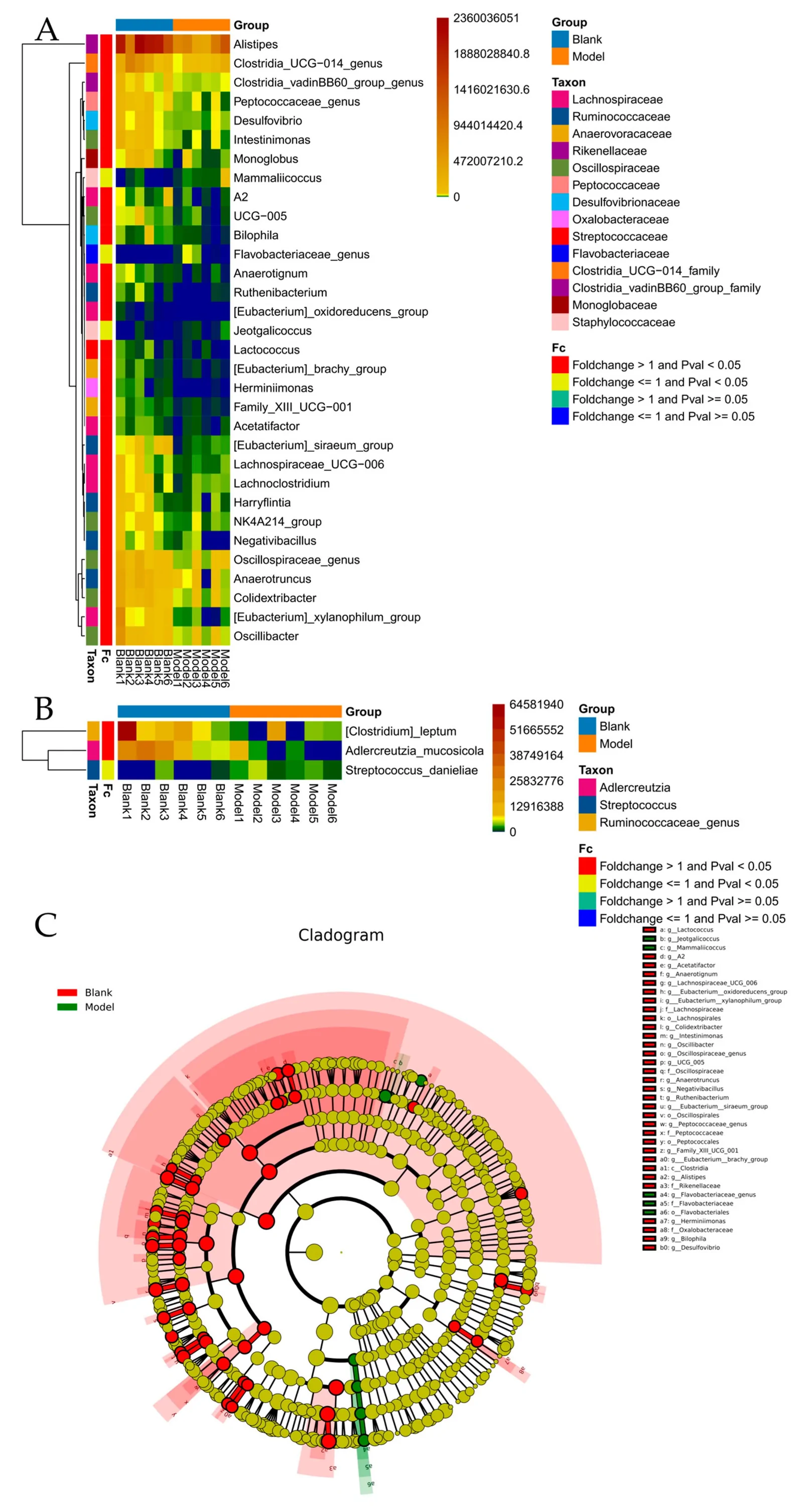
The changes in the abundance of gut microbiota after *S.* Thompson infection. (**A**) Taxonomic composition of gut microbiota at the genus level showing absolute abundance in different groups. (**B**) Taxonomic composition of gut microbiota at the species level showing absolute abundance in different groups. (**C**) Evolutionary tree diagram of LEfSe cluster analysis.

**Figure 7:**
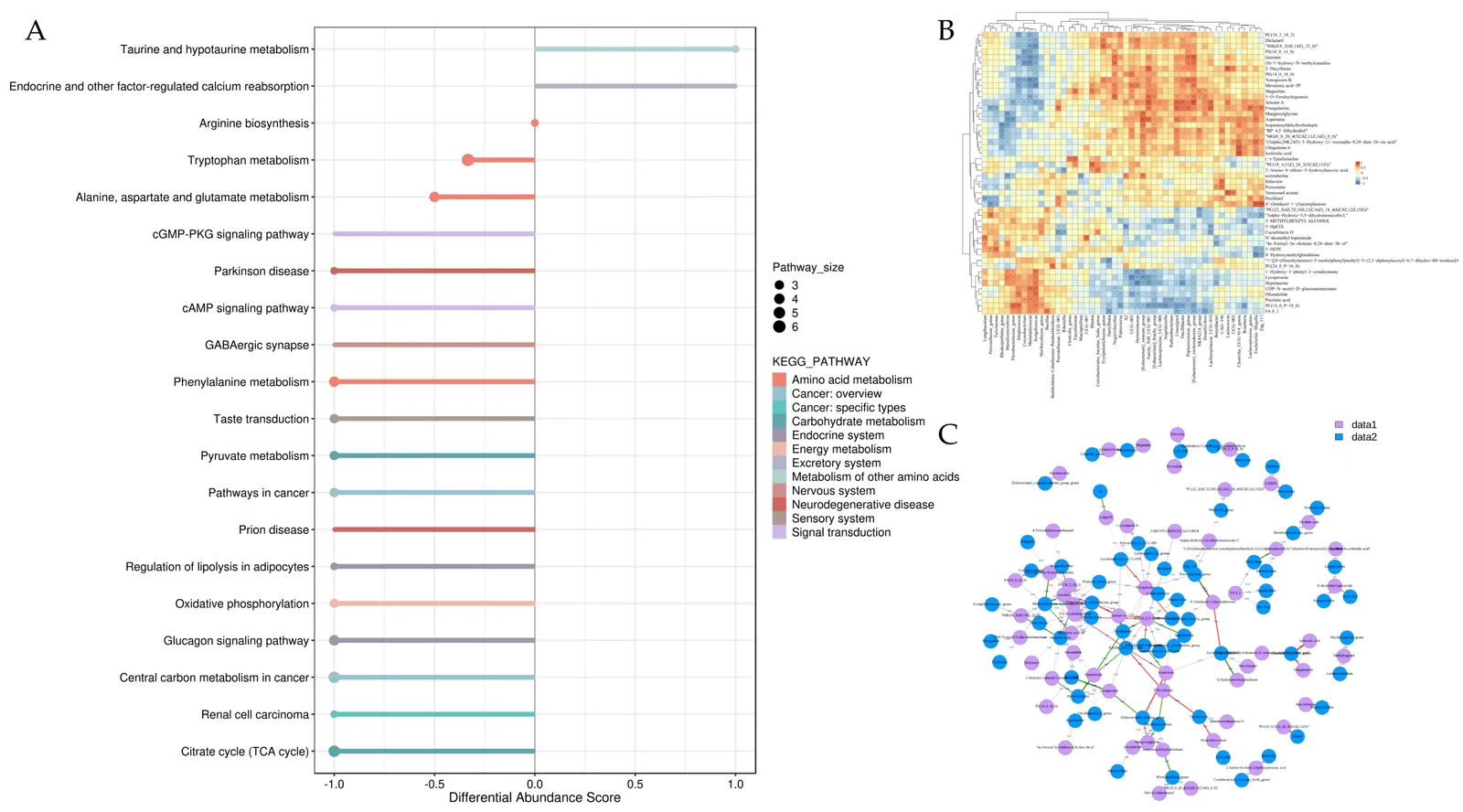
Non-targeted blood metabolomics. (**A**) KEGG enrichment bubble chart of differential metabolites. (**B**) The clustered correlation patterns between metabolites and gut microbiota. *: * (*p* < 0.05), ** (*p* < 0.01), *** (*p* < 0.001). (**C**) The network between metabolites and gut microbiota.

**Table 1:** Antimicrobial resistance rates by antimicrobial category among the seven *S.* Thompson isolates.

Antimicrobial Category	Agents	Resistant Isolates	Resistance Rate
Penicillins	AMP, PIP	C6277, C6465	2/7 (28.6%)
Non-extended-spectrum cephalosporins (first/second gen.)	CZ, CN, CXM	C6465	1/7 (14.3%)
Extended-spectrum cephalosporins (third gen.)	CAZ, CTR, CPZ	C6465	1/7 (14.3%)
Carbapenems	IPM	—	0/7 (0%)
Aminoglycosides	AMK, GEN, KAN, S	C6277, C6465	2/7 (28.6%)
Tetracyclines	TET, DO, MI	C6277, C6465	2/7 (28.6%)
Fluoroquinolones	CIP, NOR	C6465	1/7 (14.3%)
Folate pathway inhibitors	SXT	C6304, C6277, C6465	3/7 (42.9%)
Phenicols	C	C6465	1/7 (14.3%)

Note: Resistance was defined according to the CLSI M100 (30th ed., 2024) disk diffusion interpretive criteria for non-typhoidal *Salmonella*. An isolate was considered resistant to a category when it was resistant to at least one agent in that category. MDR was defined as resistance to at least one agent in three or more categories: C6277 was resistant to four categories (penicillins, aminoglycosides, tetracyclines, and folate pathway inhibitors), C6465 to eight categories (all except carbapenems), and the remaining five isolates to at most one category (C6304: folate pathway inhibitors).

## Data Availability

The datasets generated during the current study are available in the Science Data Bank repository, accessible via https://www.scidb.cn/en/anonymous/ZnVFM2V5 [50] (accessed on 9 September 2025) with doi.org/10.57760/sciencedb.27877. In particular, the full uncropped, unedited parent image has already been provided in the dataset. After the review is passed, the data will be released in time and can be publicly accessed by all users. The ARRIVE guidelines 2.0 that support the findings of this study are openly available in “Science Data Bank” via https://www.scidb.cn/en/anonymous/bk1CQkp2 (accessed on 9 September 2025) [24] with doi 10.57760/sciencedb.28731.

## Supplementary Materials

The following supporting information can be downloaded at https://www.mdpi.com/2076-2607/14/10/2229/s1: Figure S1: The changes in the abundance of gut microbiota after *S.* Thompson infection analysis; Figure S2: Non-targeted blood metabolomics analysis; Table S1: The detailed information and recovery procedures of strains; Table S2: Determination of the biofilm (BF) formation ability; Table S3: Primers and amplified conditions used for the polymerase chain reaction; Table S4: Reclassified per-isolate antimicrobial resistance matrix of the seven *S.* Thompson isolates based on the CLSI M100 interpretive criteria for Enterobacteriaceae; Table S5: CLSI M100 (30th ed., 2024) disk diffusion and MIC interpretive criteria for the 30 antimicrobial agents tested against the seven *S.* Thompson isolates. References [1,13,44,45,46,47,48,49] are cited in the Supplementary Materials.

## Abbreviations

The following abbreviations are used in this manuscript:

ESBLs	extended-spectrum β-lactamases
NTS	non-typhoidal *Salmonella*
SPIs	*Salmonella* pathogenicity islands
MDR	multiple drug resistance
PC	phosphatidylcholine
AMR	antimicrobial resistance
NB	Nutrient Broth
XLD	Xylose Lysine Desoxycholate
CLSI	Clinical and Laboratory Standards Institute
PBS	phosphate-buffered saline
PCR	polymerase chain reaction
CFU	colony-forming unit
SPF	Specific Pathogen Free
MY	lincomycin
VAN	vancomycin
PB	polymyxin B
SXT	trimethoprim/sulfamethoxazole
C	chloramphenicol
CC	clindamycin
LEV	levofloxacin
IPM	imipenem
DO	doxycycline
FFC	florfenicol
AMK	amikacin
GEN	gentamicin
KAN	kanamycin
S	streptomycin
TET	tetracycline
MI	minocycline
E	erythromycin
AZI	azithromycin
NOR	norfloxacin
CIP	ciprofloxacin
PEN	penicillin
OX	oxacillin
AMP	Ampicillin
PIP	piperacillin
CN	cephalexin
CZ	cefazolin
CXM	cefuroxime sodium
CAZ	ceftazidime
CTR	ceftriaxone
CPZ	cefoperazone
PCA	principal component analysis
PCoA	principal coordinate analysis
NMDS	non-metric Multidimensional Scaling
HACCP	Hazard Analysis and Critical Control Points
BF	biofilm
