# Supplementary material for: Biofilm-Forming and Multidrug-Resistant Salmonella Thompson Strains Isolated from Edible Cipangopaludina cathayensis: An Emerging Food Safety Concern in Southern China
Source: Microorganisms. 2026 Oct 2;14(10):2229. doi: 10.3390/microorganisms14102229 (PMC13720547; doi:10.3390/microorganisms14102229)
Supplement: Supplementary file 1 [file microorganisms-14-02229-s001.zip › microorganisms-4523940-supplementary.pdf]

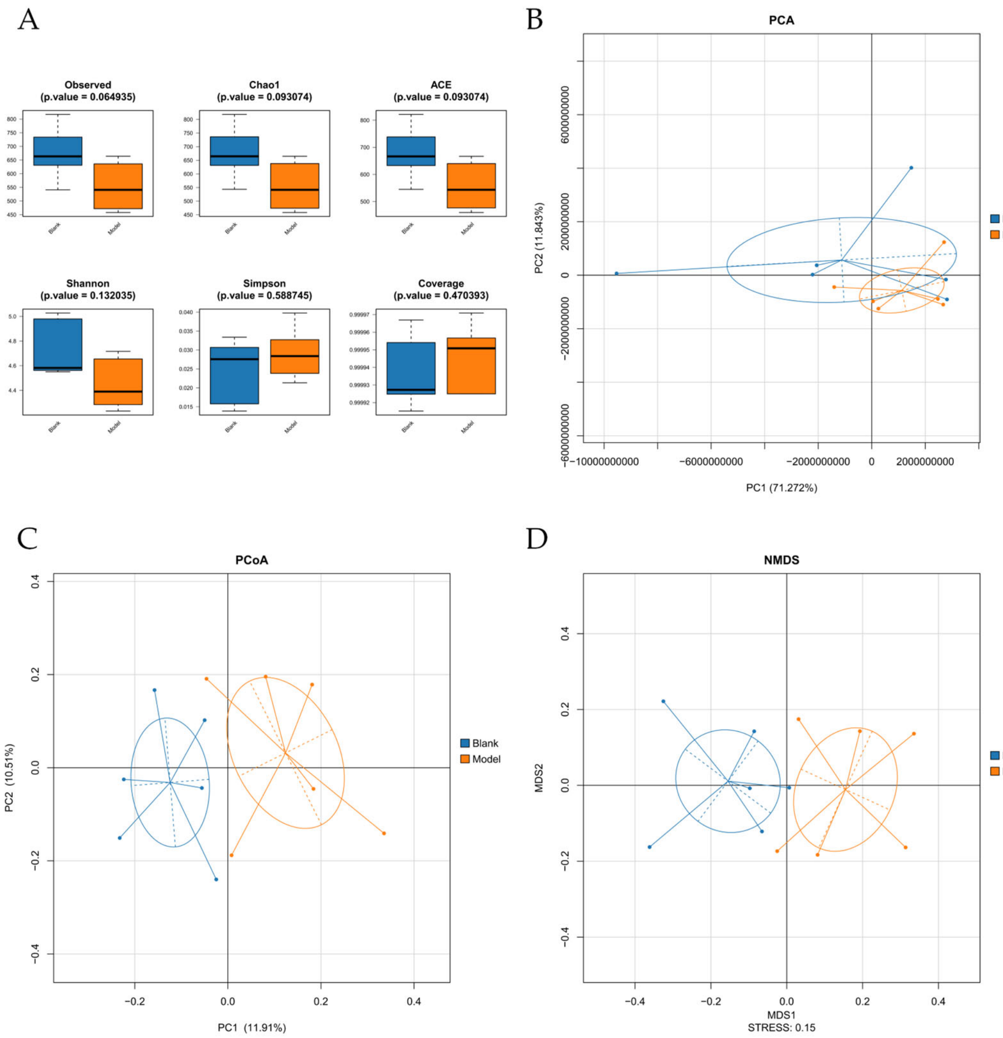

Figure S1. The changes in the abundance of gut microbiota after *S.* Thompson infection analysis. **A:** α-diversity (All data were analyzed by Kruskal-Wallis nonparametric test for intergroup differences.). **B:** Principal Component Analysis (PCA) of gut microbiota in different groups. **C:** Principal coordinate analysis (PCoA) of gut microbiota in different groups. **D:** Non-metric Multidimensional Scaling (NMDS) of gut microbiota in different groups.

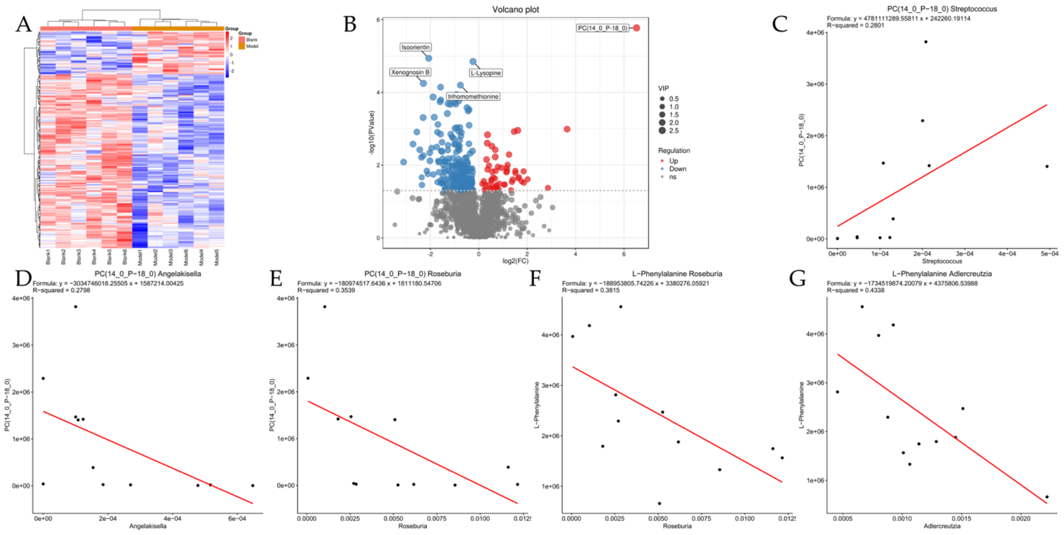

Figure S2. Non-targeted blood metabolomics analysis. **A:** The clustered heatmap of difference metabolites in different groups. **B:** Volcano Plot of difference metabolites. **C:** The strongest positive specific correlations between PC (14:0-P-18:0) and Streptococcus. **D:** The negative strongest specific correlations between PC (14:0-P-18:0) and Angelakisella. **E:** The negative strongest specific correlations between PC (14:0-P-18:0) and Roseburia. **F:** The negative strongest specific correlations between Roseburia and L-Phenylalanine. **G:** The negative strongest specific correlations between L-Phenylalanine and Adlercreutzia.

Table S1. The detailed information and recovery procedures of strains.

Strains	Time	Isolated city	Recovered procedures
C6304	2021.02.10	Laibin, the Hongshui River	Under aseptic conditions, a 1 mL aliquot of the isolates cultures was inoculated into 100 mL portions of Nutrient Broth (NB) incubated at 37 °C for 18 h. Followed by inoculating into Xylose Lysine Desoxycholate (XLD) agar plates. After a 24 h incubation period at 37 °C, potential *Salmonella* colonies were then individually inoculated into both triple sugar iron and urea-agar slants, incubated for an additional 24 h at 37 °C. The serotypes of the isolates were determined by the slide agglutination test using the Kauffman-White serotyping method with the anti-*Salmonella* serum [1].
C6458	2021.02.09	Nanning, the Zhupaichong river	
C6469	2021.02.10	Nanning, the Kelijiang river	
C6277	2021.02.09	Laibin, the Beizhijiang river	
C6465	2021.02.10	Nanning, the Xinxujiang river	
C6273	2022.07.18	Laibin, the Beizhijiang river	
C6272	2021.05.06	Laibin, the Hongshui River	

Note: Because the isolates were collected at different time points between 2021 and 2022 that were not perfectly matched between the two cities, sampling-time differences may represent a potential confounding factor in the comparison of city-level patterns.

Table S2. Determination of the biofilm (BF) formation ability.

BF formation ability	OD value	Procedures of crystal violet staining method
strong BF production	4 ODc < OD	The activated bacterial solution was inoculated into 96-well microtiter plates with a final concentration of 1×10^6 CFU/mL and then incubated for 48 h until the biofilm attained maturity. At first, most of the bacterial solution was decanted and washed twice with phosphate-buffered saline (PBS) to remove any non-adherent cells. Subsequently, methanol was added for fixation for 15 minutes, after which the methanol was discarded and allowed to evaporate completely. Then, crystal violet staining was performed for 5 min, after which the crystal violet was discarded. After being washed with clear water until the effluent was colorless, anhydrous ethanol was added for decolorization (Chen, et al., 2023 [13]). Finally, the absorbance was quantified at 570 nm by the microplate reader.
moderate BF production	2ODc < OD ≤ 4ODc	
weak BF production	ODc < OD ≤ 2ODc	
no BF production	OD ≤ ODc	

Note: The mean optical density (OD) value of the negative control group was recorded as ODc. The classification criteria were based on previous studies and were chosen to facilitate comparison with other BF formation bacteria. The *Salmonella* strains that exhibit the highest OD values within the strong BF production group were selected for subsequent experiments in this study, as they were deemed to have the greatest potential for BF formation and could provide valuable insights into the mechanisms of BF development in *Salmonella* species.

Table S3. Primers and amplified conditions used for the polymerase chain reaction.

**Target** *gene*	**Sequence (5′ - 3′)**	**Amplifcation product (bp)**	**Amplified conditions of PCR**	**Reference**
**Virulence** ***genes***				
stn	F: AGCGTTCAGGTACAGATTCAACA<br>R: AAATTCGTAACCCGCTCTCGT	341	2 min initial denaturation at 94 °C followed by 32 cycles of 94 °C for 30s, 59 °C for 30s and 72 °C for 35s and a final incubation at 72 °C for 2 min.	[44]
fimA	F: AGACCGCCAGCAAATTAGTGT<br>R:TGACCTCTACTATTGCGAGTCTG	321		[44]
virK	F: CAAAGGCTATTCTGGAACACT<br>R: CACCTTTCTTAACGCCGTTT	519	2 min initial denaturation at 94 °C followed by 32 cycles of 94 °C for 30s, 55 °C for 30s and 72 °C for 35s and a final incubation at 72 °C for 2 min.	[44]
invA	F: TGTTCGTCATTCCATTACCT<br>R: ATCGGCATCAATACTCATCT	395		[44]
sseL	F: GATTACTTTATATGGCCGGTT<br>R: AAATAGACCACAGCCGTTG	527	2 min initial denaturation at 94 °C followed by 32 cycles of 94 °C for 30s, 54 °C for 30s and 72 °C for 35s and a final incubation at 72 °C for 2 min.	[44]
mgtC	F: CGCACATTCATCCCTTCGC<br>R: AAATTTACTGGCCGCTATGCT	231		[44]
siiE	F: CGCCTCCAACCATAAAGCTC<br>R: CGATACCACGACAAAGGCAT	800	2 min initial denaturation at 94 °C followed by 32 cycles of 94 °C for 30s, 57 °C for 30s and 72 °C for 35s and a final incubation at 72 °C for 2 min.	[44]
sopB	F: ACTTATTGCAGCACGCACTCA<br>R: CAGGGCGTAAATCATTGCCT	782		[44]

	A			
**β-lactamase** ***genes***				
bla$_{TEM-1}$	F: GTGCTGCCATAACCATGAGT<br>R: CTGCAACTTTATCCGCCTCC	282	2 min initial denaturation at 94 °C followed by 30 cycles of 94 °C for 30s, 55 °C for 30s and 72 °C for 35s and a final incubation at 72 °C for 2 min.	[1]
bla$_{CMY-2}$	F: TTACTGCGTTATTTATTCCCAC<br>R: CGCCCAAAATGACTTTAGCAA	339	2 min initial denaturation at 94 °C followed by 30 cycles of 94 °C for 30s, 52 °C for 30s and 72 °C for 35s and a final incubation at 72 °C for 2 min.	[45]
bla$_{CTX-M}$	F: AAGTGAAAGCGAACCGAATCT<br>R: ATTGCCCGAGGTGAAGTG	309	5 min initial denaturation at 95 °C followed by 35 cycles of 95 °C for 30s, 59 °C for 40s and 72 °C for 60s and a final incubation at 72 °C for 10 min.	[45]
bla$_{PSE-1}$	F: TGCTTCGCAACTATGACTAC<br>R: AGCCTGTGTTTGAGCTAGAT	438	2 min initial denaturation at 94 °C followed by 30 cycles of 94 °C for 30s, 55 °C for 30s and 72 °C for 35s and a final incubation at 72 °C for 2 min.	[1]
bla$_{SHV}$	F: CCGGGTTATTCTTATTTGTCGCT<br>R: TAGCGTTGCCAGTGCTCG	831	5 min initial denaturation at 95 °C followed by 35 cycles of 95 °C for 20s, 59 °C for 40s and 72 °C for 60s and a final incubation at 72 °C for 5 min.	[46]
bla$_{OXA}$	F: GTCTTTC(A/G)AGTACGGCATTA<br>R: GATTTTCTTAGCGGCAACTTA	822	2 min initial denaturation at 93 °C followed by 35 cycles of 93 °C for 60s, 55 °C for 60s and 72 °C for 60s and a final incubation at 72 °C for 2 min.	[47]
bla$_{NDM}$	F: GGTTTGGCGATCTGGTTTTC<br>R: CGGAATGGCTCATCACGATC	621	4 min initial denaturation at 95 °C followed by 25 cycles of 94 °C for 10s, 52 °C for 30s and 72 °C for 60s and a final incubation at 72 °C for 5 min.	[48]
Biofilm *genes*				
csgA	F: AATAGTTCCGGCCCGGACTC<br>R: ACCGCCGTATTGGCCGACAGT	252	5 min initial denaturation at 95 °C followed by 40 cycles of 95 °C for 10s, 58 °C for 30s and 72 °C for 30s and a final incubation at 72 °C for 5 min.	[49]
csgD	F: AACACGTGGTCAGCGGATTACAG<br>R: CGACCTCGCGATTTCATTATTAGA	178		

Note: All the PCR products were directly sequenced by BGI Co. Ltd. (Beijing, China) to confirm the accuracy of target *genes* of PCR.

Table S4. Reclassified per-isolate antimicrobial resistance matrix of the seven *S.* Thompson isolates based on the CLSI M100 interpretive criteria for Enterobacteriaceae.

Antimicrobial agent	Class	C6304	C6458	C6469	C6277	C6465	C6273	C6272
Lincomycin (MY)+	Lincosamide	R	R	R	R	R	R	R

Clindamycin (CC)+	Lincosamide	R	R	R	R	R	R	R
Vancomycin (VAN)+	Glycopeptide	R	R	R	R	R	R	R
Erythromycin (E)+	Macrolide	R	R	R	R	R	R	R
Oxacillin (OX)+	Penicillin	R	R	R	R	R	R	R
Penicillin (PEN)	Penicillin	I	I	I	R	R	I	I
Ampicillin (AMP)	Penicillin	S	S	S	R	R	S	S
Piperacillin (PIP)	Penicillin	S	S	S	R	R	S	S
Cephalexin (CN)	Cephalosporin	I	I	I	I	R	I	I
Cefazolin (CZ)	Cephalosporin	S	S	S	I	R	S	S
Cefuroxime sodium (CXM)	Cephalosporin	S	S	S	S	R	S	S
Ceftazidime (CAZ)	Cephalosporin	S	S	S	S	R	S	S
Ceftriaxone (CTR)	Cephalosporin	S	S	S	S	R	S	S
Cefoperazone (CPZ)	Cephalosporin	S	S	S	I	R	S	S
Imipenem (IPM)	Carbapenem	S	S	S	S	S	S	S
Amikacin (AMK)	Aminoglycoside	S	S	S	S	S	S	S
Gentamicin (GEN)	Aminoglycoside	S	S	S	S	S	S	S
Kanamycin (KAN)	Aminoglycoside	S	S	S	S	S	S	S
Streptomycin (S)	Aminoglycoside	S	S	S	R	R	S	S
Doxycycline (DO)	Tetracycline	S	I	S	R	R	I	I
Tetracycline (TET)	Tetracycline	S	S	S	R	R	S	S
Minocycline (MI)	Tetracycline	S	S	I	I	I	I	S
Azithromycin (AZI)	Macrolide	S	S	S	S	R	S	S
Levofloxacin (LEV)	Fluoroquinolone	S	S	I	I	R	I	I
Norfloxacin (NOR)	Fluoroquinolone	S	S	S	I	R	S	S
Ciprofloxacin (CIP)	Fluoroquinolone	S	S	S	S	R	S	S
Trimethoprim/sulfamethoxazole (SXT)	Folate-pathway inhibitor	R	S	S	R	R	S	S
Chloramphenicol (C)	Phenicol	S	S	S	S	R	S	S
Florfenicol (FFC)	Phenicol	S	S	I	I	R	I	I
Polymyxin B (PB)	Polymyxin	S	S	S	S	S	S	S

Note: Ten antimicrobial agents lacked validated CLSI M100 criteria for non-typhoidal *Salmonella*. Susceptibility categories (R, I, S) were assigned per CLSI M100 Enterobacteriaceae breakpoints. Multidrug resistance (MDR) was defined as nonsusceptibility to ≥3 antimicrobial classes with applicable CLSI M100 criteria.

Table S5. CLSI M100 (30th ed., 2020) disk-diffusion and MIC interpretive criteria for the 30 antimicrobial agents tested against the seven *S.* Thompson isolates.

Antimicrobial agent	Class	Disk content	Zone diameter (mm): S / I / R	MIC (μg/mL): S / I / R	Applicable to non-typhoidal *Salmonella*
Ampicillin (AMP)	Penicillin	10 μg	≥17 / 14–16 / ≤13	≤8 / 16 / ≥32	Yes
Piperacillin (PIP)	Penicillin	100 μg	≥21 / 18–20 / ≤17	≤16 / 32–64 / ≥128	Yes
Penicillin (PEN)	Penicillin	—	No criteria	No criteria	No (no criteria for Enterobacteriaceae)

Oxacillin (OX)	Penicillin	—	No criteria	No criteria	No (no criteria for Enterobacteriaceae)
Cefazolin (CZ)	Cephalosporin (1st gen.)	30 µg	≥23 / 20–22 / ≤19	≤2 / 4 / ≥8	Criteria exist; 1st-gen. cephalosporins not reported as susceptible for *Salmonella*
Cephalexin (CN)	Cephalosporin (1st gen.)	(via cefazolin surrogate)	≥15 (U, surrogate) / — / ≤14	≤16 / — / ≥32 (surrogate)	Criteria exist via surrogate; not reported as susceptible for *Salmonella*
Cefuroxime sodium (CXM)	Cephalosporin (2nd gen.)	30 µg	≥18 / 15–17 / ≤14	≤8 / 16 / ≥32	Criteria exist; 2nd-gen. cephalosporins not reported as susceptible for *Salmonella*
Ceftazidime (CAZ)	Cephalosporin (3rd gen.)	30 µg	≥21 / 18–20 / ≤17	≤4 / 8 / ≥16	Yes
Ceftriaxone (CTR)	Cephalosporin (3rd gen.)	30 µg	≥23 / 20–22 / ≤19	≤1 / 2 / ≥4	Yes
Cefoperazone (CPZ)	Cephalosporin (3rd gen.)	75 µg	≥21 / 16–20 / ≤15	≤16 / 32 / ≥64	Yes (report as resistant if ESBL-positive)
Imipenem (IPM)	Carbapenem	10 µg	≥23 / 20–22 / ≤19	≤1 / 2 / ≥4	Yes
Amikacin (AMK)	Aminoglycoside	30 µg	≥17 / 15–16 / ≤14	≤16 / 32 / ≥64	Criteria exist; aminoglycosides not reported as susceptible for *Salmonella*
Gentamicin (GEN)	Aminoglycoside	10 µg	≥15 / 13–14 / ≤12	≤4 / 8 / ≥16	Criteria exist; aminoglycosides not reported as susceptible for *Salmonella*
Kanamycin (KAN)	Aminoglycoside	30 µg	≥18 / 14–17 / ≤13	≤16 / 32 / ≥64	Criteria exist; aminoglycosides not reported as susceptible for *Salmonella*
Streptomycin (S)	Aminoglycoside	10 µg	≥15 / 12–14 / ≤11	No MIC criteria	Criteria exist; aminoglycosides not reported as susceptible for *Salmonella*
Tetracycline (TET)	Tetracycline	30 µg	≥15 / 12–14 / ≤11	≤4 / 8 / ≥16	Yes
Doxycycline (DO)	Tetracycline	30 µg	≥14 / 11–13 / ≤10	≤4 / 8 / ≥16	Yes
Minocycline (MI)	Tetracycline	30 µg	≥16 / 13–15 / ≤12	≤4 / 8 / ≥16	Yes
Erythromycin (E)	Macrolide	—	No criteria	No criteria	No (no criteria for Enterobacteriaceae)
Azithromycin (AZI)	Macrolide	15 µg	≥13 / — / ≤12	≤16 / — / ≥32	Only for *Salmonella* Typhi; not for non-typhoidal *Salmonella*
Ciprofloxacin (CIP)	Fluoroquinolone	5 µg	≥31 / 21–30 / ≤20 (*Salmonella*)	≤0.06 / 0.12–0.5 / ≥1 (*Salmonella*)	Yes (*Salmonella*-specific criteria)
Levofloxacin (LEV)	Fluoroquinolone	—	No disk criteria for *Salmonella*	≤0.12 / 0.25–1 / ≥2 (*Salmonella*)	MIC criteria only for *Salmonella*; no disk criteria
Norfloxacin (NOR)	Fluoroquinolone	10 µg	≥17 / 13–16 / ≤12	≤4 / 8 / ≥16	Yes
Trimethoprim/ sulfamethoxazole (SXT)	Folate-pathway inhibitor	1.25/ 23.75 µg	≥16 / 11–15 / ≤10	≤2/38 / — / ≥4/76	Yes
Chloramphenicol (C)	Phenicol	30 µg	≥18 / 13–17 / ≤12	≤8 / 16 / ≥32	Yes
Florfenicol (FFC)	Phenicol	—	No criteria	No criteria	No (veterinary agent; no criteria in M100)
Polymyxin B (PB)	Polymyxin	—	No disk criteria	≤2 / — / ≥4 (broth	No disk-diffusion criteria; broth microdilution only

				microdilution only)	
Lincomycin (MY)	Lincosamide	—	No criteria	No criteria	No (no criteria for Enterobacteriaceae)
Clindamycin (CC)	Lincosamide	—	No criteria	No criteria	No (no criteria for Enterobacteriaceae)
Vancomycin (VAN)	Glycopeptide	—	No criteria	No criteria	No (no criteria for Enterobacteriaceae)

Note: Disk diffusion and minimum inhibitory concentration (MIC) interpretive criteria follow CLSI document M100. Zone diameter (mm) and MIC (µg/mL) breakpoints are categorized as susceptible (S), intermediate (I), or resistant (R). Agents marked with "+" lack CLSI-endorsed criteria for non-typhoidal *Salmonella* and are included for descriptive purposes only.
